# The role of NH_4_Cl and cysteine proteases in Human Papillomavirus type 16 infection

**DOI:** 10.1186/1743-422X-6-109

**Published:** 2009-07-20

**Authors:** Sarah A Dabydeen, Patricio I Meneses

**Affiliations:** 1 Department of Microbiology and Immunology, H.M. Bligh Cancer Research Laboratory, School of Graduate and Postdoctoral Studies, Rosalind Franklin University of Medicine and Science, North Chicago, IL, USA; 2 Department of Microbiology and Immunology, H.M. Bligh Cancer Research Laboratory, Chicago Medical School, Rosalind Franklin University of Medicine and Science, North Chicago, IL, USA

## Abstract

**Background:**

The infectious pathway of the non-enveloped Human Papillomavirus Type 16 (HPV16) includes binding to the cell surface, clathrin-mediated endocytosis, and penetration into an endosome. HPV16 infection was shown to decrease in the presence of the lysosomotrophic neutralizing agent ammonium chloride (NH_4_Cl). NH_4_Cl neutralizes acidic endo-lysosome compartments, thus suggesting that pH was responsible for PV capsid conformational changes leading endosome escape.

**Results:**

However, our data suggested that NH_4_Cl blocked infection by preventing the movement of PV viral particles from the early endosome to the caveosome as was shown for JC virus [1,2]. We have confirmed that HPV 16 infection requires the trafficking of reporter-virions to the caveosome as is the case for BPV1 [3,4]. In this manuscript we propose that the observed decrease in infection of PV in the presence of NH_4_Cl was due to a loss of movement of reporter-virions to caveosomes. We also demonstrate that cysteine proteases are involved in the infectious process, and that cathepsin B treatment of viral particles was shown to overcome the block of infection observed in the presence of furin inhibition. We confirmed the need for cathepsin B in HPV16 infection using cathepsin B null mouse embryonic fibroblasts.

**Conclusion:**

We present data that suggest HPV16 infection is in part mediated by cysteine proteases, and that NH_4_Cl blocks the intracellular trafficking of infectious viral particles. To our knowledge this is the first demonstration that cysteine proteases influence the infection of a non-enveloped virus.

## Background

Human Papillomaviruses (HPVs) are non-enveloped DNA viruses that can infect the skin and mucous membranes. HPVs are known to cause cutaneous, cervical, and respiratory warts and lesions [5-7]. The capsid of HPVs is made of two virally encoded structural proteins L1 and L2 [8-10]. The major capsid protein L1 is primarily involved in attachment of the virus to the plasma membrane, while the minor capsid protein L2 functions in viral genome trafficking and encapsidation [11-15].

The infectious process begins via virion attachment to the cell surface through breaks in the skin. Although the virion-cell binding process is still unclear it is thought to occur by initial binding of the L1 protein on the virion capsid to heparan sulfate (a cell surface proteoglycan), followed by binding to a secondary receptor, putatively an integrin complex [16-18]. α6β4 has been shown to be able to mediate cell binding in studies showing that antibodies against α6 could block virion binding to the epithelial cells CV-1 and HaCaT keratinocytes [19]. However, α6β4 integrin may not be a necessary requirement for infection since studies also indicate that some PVs can infect cells such as BO-SV keratinocytes that lack this complex [20]. After attachment to the cell surface the HPV16 virion is internalized via a mechanism that begins with clathrin mediated endocytosis [2,21,22]. N-terminus cleavage of L2 by furin, a calcium dependent serine endoprotease found at the plasma membrane, Golgi and endosomes, has been suggested to be required for infection [23,24]. Our data suggests that after trafficking to the endosome, the reporter-virions may follow either an infectious route or a noninfectious route ([3,4]). In the infectious route, reporter-virions are moved to the caveolin-1 intracellular sorting pathway. This caveolin-1 pathway was shown to be necessary for infection, as infection is blocked in cells where caveolin-1 protein levels were reduced using siRNA against caveolin-1 ([3,4]). After entering the caveosome, the virion was shown to traffic in an L2-mediated event to a region where it colocalized with the endoplasmic reticulum (ER) t-SNARE syntaxin 18 and the ER chaperone calnexin and ERp29 ([3,4,11,14]). The non-infectious pathway results in trafficking from the endosome to the lysosome where reporter-virions may be processed for degradation by the cell. This latter pathway was shown using a non-infectious L2 mutant virus and neutralizing antibodies [3]. It has been shown that ammonium chloride (NH_4_Cl) blocks infection of Bovine Papillomavirus Type 1 (BPV1), a PV with similar kinetics to HPV16 [2]. NH_4_Cl neutralized the acidic endo-lysosome compartments suggesting that pH was responsible for PV capsid conformational changes leading to viral genome release. However, our data presented in this manuscript suggested that ammonium chloride blocked infection by preventing the movement of viral particles from the early endosome to the caveosome as was also shown for JC virus [1]. In this manuscript we show that cysteine proteases and not pH may be responsible for changes leading to infection.

Cysteine proteases function as intracellular and extracellular molecules [25]. The cysteine protease cathepsin B is associated with caveolae. Caveolae are defined as small invaginations of the plasma membrane associated with lipid rafts that contain caveolin-1 [26,27]. Similar to caveolae, endo-lysosomal compartments within cells contain cathepsin B but in addition have cathepsin L. Both of these cathepsins are zymogens (pro-forms) that are cleaved into their active form [28,29]. The exact mechanism of activation is not well understood however, activation of pro-cathepsin B may occur by S100A10, a protein found in caveolae, while activation of pro-cathepsin L may occur by heparan sulfate, a possible receptor for PV [27,30]. In addition to caveolae and endosomes, cathepsins have been found to be associated with lipid rafts, suggesting that cathepsins may be needed upon internalization to break apart matrices on viral surfaces [25].

Cathepsins B and L have been implicated in the mechanism of binding, entry and disassembly of several enveloped viruses. In the case of binding, treating Ebolavirus reporter-virions with cathepsin L enhanced infectivity by cleaving and removing a highly glycosylated mucin domain in the Ebolavirus glycoprotein and resulted in increased binding suggesting that cathepsins are indeed present on the cell membrane [31]. Fusion of enveloped viruses such as Nipah, Hendra, SARS Coronavirus and Murine Coronavirus Mouse Hepatitis Virus has been shown to be dependent on cathepsins B and L. In Nipah virus, both cathepsin B and L were shown to cleave a membrane fusion protein required for virus-cell and cell-cell membrane fusion. Cleavage of the viral membrane fusion protein into the correct size was necessary for maturation into a fusogenic form. Cathepsin B was shown to cleave the fusion protein in a cell-free system into two fragments but the smaller of these fragments migrated slower than fragments produced during the cleavage that occurs in infection, suggesting that the fusion protein was not cleaved at the correct size. Cathepsin L was able to cleave the fusion protein into fragments of the correct size in the cell-free system suggesting that although cathepsin B and L are catalytically similar, they may have distinct target/sequence specificity [32]. Similar to Nipah virus, cathepsin L was involved in cleaving the Hendra virus fusion protein into an active heterodimer [33]. In SARS Coronavirus infection, cathepsin L was needed to cleave the spike protein, one of four major structural proteins, into two subunits: one having a high binding affinity to the receptor and the other mediating fusion of viral and cellular membranes [34]. The requirement of cathepsins for fusion was also shown in Murine Coronavirus Mouse Hepatitis Virus (MHV), where proteolysis by cathepsin B and L was necessary for cleavage of the MHV-2 spike protein [35]. In addition, proteolysis by cathepsins was shown to be important for disassembly of Reovirus. Using mouse embryonic fibroblasts derived from cathepsin B or L deficient and wild type mice, studies show that Reovirus disassembly was prohibited in the absence of cathepsins B and L [36].

In this manuscript, we show a role for cathepsin B that may be important for HPV16 infection. It is, to our knowledge, the first description of the role of cysteine proteases on a non-enveloped virion.

## Results

### Cysteine protease inhibitors inhibit HPV16 infection

**Figure 1:**
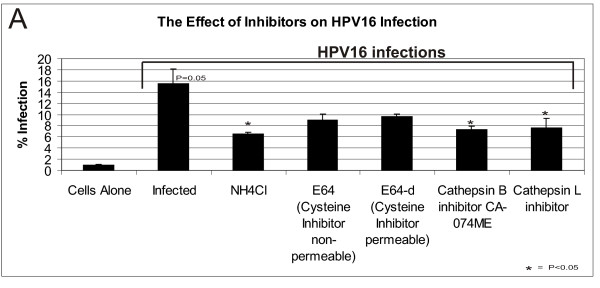
**HPV16 infection is reduced in the presence of cysteine protease inhibitors**. Infection of 293 cells alone, with HPV 16 DsRed reporter-virions (mock control, Infected), or in the presence of: 20 mM of lysosome neutralizing agent NH_4_Cl; 10 μM of a non-permeable cysteine protease inhibitor E64; 10 μM of the permeable cysteine protease inhibitor E64-d; 6 μM of the permeable intracellular cathepsin B inhibitor CA-074ME; or 10 μM of the cathepsin L inhibitor. Infection was analyzed and compared 48 hours post binding by FACS of DSRED expression. Inhibitors were present for the duration of infection. Cells alone were analyzed for background fluorescence. Statistics were analyzed by 1 tailed t-test and found to be significant at P < 0.05.

To determine the role of cysteine proteases on HPV16 infection, we performed infections of 293 cells in the presence of protease inhibitors at non-toxic concentrations [see Additional file 1] and compared them to the loss of infection in the presence of the lysosome pH neutralizing agent ammonium chloride (NH_4_Cl). The dose dependent concentration effects of the inhibitors on HPV16 infection are also shown [see Additional file 2]. Infection by HPV16 DsRed reporter-virions was determined by FACS analysis of DsRed fluorescent cells after 48 hours. Compared to cells infected in the absence of inhibitor (Fig 1, infected bar), we observed a 61.45% decrease in infection the presence of NH_4_Cl (Fig 1, NH_4_Cl bar), a 41.38% and 37.30% decrease in the presence of permeable and non-permeable broad targeting cysteine protease inhibitors (Fig. 1, E64 and E64-d bar, respectively), and a statistically significant decrease in infectivity of 53.75% and 51.11% in the presence of specific cathepsin B or cathepsin L inhibitors (Fig 1 cathepsin B and cathepsin L bars respectively) (p < 0.05 1-tail t test). The experiment was repeated in HaCaT cells and shows that in the presence of either NH_4_Cl or the cysteine protease inhibitor E64, a decrease in infection is observed compared to cells that were infected in the absence of inhibitor [see Additional file 3]. These data suggested a role for cysteine proteases in HPV16 infectivity.

### Ammonium chloride prevents HPV16 trafficking to caveosomes

**Figure 2:**
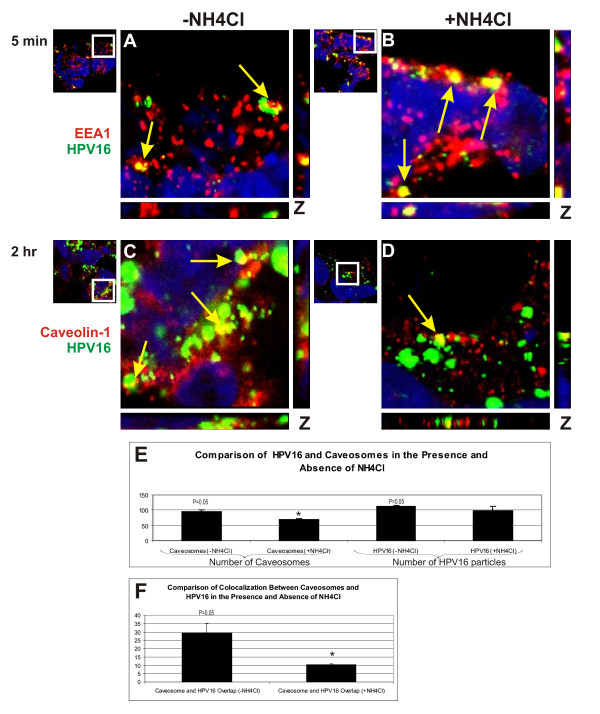
**Ammonium Chloride blocks the trafficking of HPV16 to caveosomes**. Infection of 293 cells alone in the absence (A and C) or presence (B and D) of 20 mM of lysosome neutralizing agent NH_4_Cl. Cells were harvested after 5 minutes post binding for early endosome staining with goat anti- EEA1 (A and B), or 2 hours post binding for caveosome staining with goat anti-caveolin 1 (C and D). HPV16 particles were stained using a monoclonal anti-HPV16 L1 antibody H16V.5 (A-D). Images are shown with z-stacks obtained by confocal microscopy. The number of caveosomes (E), HPV16 reporter-virions (E), and colocalization between HPV16 and caveosomes (F) in the absence and presence of NH_4_Cl were counted using stereology software.

In the presence of NH_4_Cl HPV16 infection levels were shown to decrease. To determine if NH_4_Cl was able to block the movement of HPV16 reporter-virions from early endosomes to caveosomes, we infected 293 cells in the presence or absence of 20 mM NH_4_Cl. Infection by GFP-Reporter HPV16 reporter-virions was assessed by confocal microscopy and stereology counts. Data was collected at 5 minutes post binding for early endosome staining (with goat anti- EEA1, Fig. 2A and 2B, red) and 2 hours post binding for caveolin-1 staining (with goat anti-caveolin 1, Fig 2C and 2D, red). HPV16 particles were stained using a monoclonal anti-HPV16 L1 antibody H16V.5 (Fig 2A–D, green). In the absence (Fig 2A) and presence of NH_4_Cl (Fig 2B) overlap is observed between HPV16 (Fig 2, green) and the early endosomes (Fig 2A and 2B red) as indicated by the yellow color (Fig 2A and 2B, yellow). We observed a decrease in colocalization between HPV16 (Fig 2 green) and caveolin-1 staining (Fig 2C and 2D, red) in the presence of NH_4_Cl as compared to the absence of NH_4_Cl (compare Fig 2D to Fig 2C, yellow). A comparison of the percent of HPV16 reporter-virions overlapping with caveolin-1 in the absence or presence of NH_4_Cl by stereology showed a statistical significant change. The percent of HPV16 reporter-virions colocalizing with caveosomes decreased by 65.52% in the presence of NH_4_Cl (Fig 2F Caveosomes and HPV16 overlap -NH_4_Cl, and Caveosome and HPV16 overlap + NH_4_Cl). This 65.52% decrease in colocalization was statistically significant at p < 0.05 1 tailed T test. We did observe a drop in the overall number of caveosomes, and no changes in the number of internalized viral particles (Fig 2E). The decrease in HPV16 infectivity was not due to the effects of NH_4_Cl on the HPV16 reporter-virions [see Additional file 4]. These data suggest that NH_4_Cl can influence that formation of caveosomes and of the movement of HPV16 reporter-virions into caveosomes similar to JC virus.

### Cathepsin B treatment of HPV16 increases infection levels

**Figure 3:**
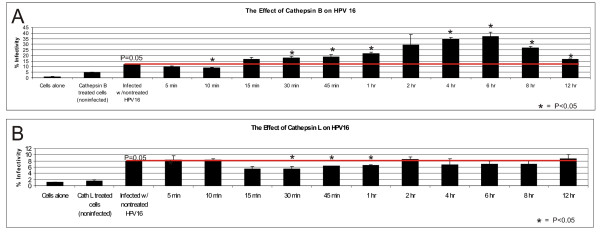
**Cathepsin B treatment of HPV16 increases infection**. HPV16 DsRed reporter-virions were incubated with (A) 37 pM cathepsin B protease or (B) 37 pM cathepsin L protease for various amounts of time. 293 cells were infected with protease treated reporter-virions or infected with untreated reporter-virions (control). Infections were analyzed by flow cytometry for DsRed expression 48 hours post binding. 293 cells alone were used as control for background fluorescence. Statistics were analyzed by 1 tailed t-test and found be significant at P < 0.05.

Because cysteine protease inhibitors reduced infection, we wanted to determine if treating purified HPV16 reporter-virions with cysteine proteases influenced infection. HPV16 reporter-virions were treated with cathepsin B or cathepsin L protease for various amounts of time at 37°C, added to cells, and infections were analyzed by flow cytometry after 48 hours (for DsRED expression). Treatment of HPV16 with cathepsin B protease for 15 minutes increased infectivity compared to untreated reporter-virions (Fig 3A, 15 min bar vs. infected/w nontreated HPV16 bar) and was observed to increase and peak at 6 hours to 3.09 fold as compared to non-treated HPV16 infections (Fig 3A, 6 hr bar vs. infected/w nontreated HPV16 bar). This increase in infection can be considered both statistically and physiologically significant since the amount of infection is 3 times greater than in the control. A lesser increase in infection was observed after 8 and minimal after 12 hrs (Fig 3A 8, 12 hr bars). Cathepsin L protease treatment of HPV16 did not enhance infection levels above untreated reporter-virions (Fig 3B red bar). These data suggested that treating HPV16 reporter-virions with cathepsin B protease enhanced infection whereas treatment with cathepsin L did not have an affect. Experiments were repeated with different reporter virion preparations with similar results. HPV16 reporter-virions are known to require a maturation step at 37°C [37]. We incubated already matured reporter-virions at 37°C throughout the time course to ensure that any increase in infectivity observed was not due reporter-virions that were partially matured further maturing at 37°C (and thus more efficient infection) [see Additional file 5]. Our results indicated that the additional 37°C incubation process did not significantly change infection levels suggesting that reporter-virions were matured, i.e., no further virion maturation occurred during the additional 37°C incubation (p < 0.05, 1-tail t test). Experiments in which cells were treated with cathepsin protease prior to infection showed no changes in infection (data not shown).

### Protease treatment of HPV16 increases binding

**Figure 4:**
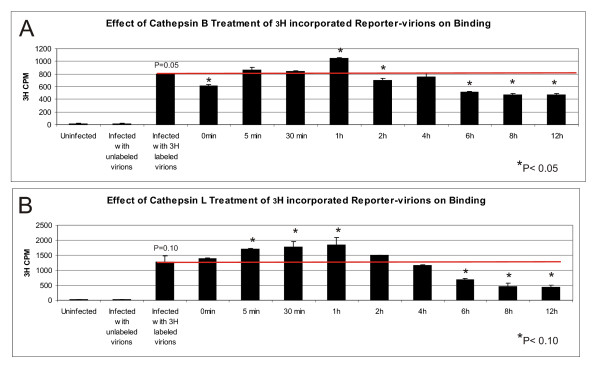
**Cathepsin B and L treatment of HPV16 increases binding**. Reporter virus binding was compared in 293 cells incubated with ^3^H labeled HPV16 reporter-virions treated for various amounts of time with (A) 37 pM cathepsin B protease or (B) 37 pM cathepsin L protease. Amount of binding was quantified by scintillation counter for the presence of ^3^H. Statistics were analyzed by 1 tailed t-test and found to be significant at P < 0.05 for (A) and P < 0.10 for (B).

In order to determine if the increase in infection observed after protease treatment of reporter-virions was due to changes in total virus binding, tritium radio-labelled HPV16 (^3^H HPV16) binding studies were performed. ^3^H HPV16 reporter-virions were incubated with cathepsin B or cathepsin L protease for various amounts of time, added to cells and allowed to bind at 4°C for 2 hours before being thoroughly washed, harvested and fixed for analysis by scintillation counter. Treatment of HPV16 reporter-virions with cathepsin B protease for 1 hour increased binding by 1.3 fold compared to the non- protease treated reporter-virions (Fig. 4A, 1 hr bar) (p < 0.05, 1-tail t test). Cathepsin L protease treatment up to 1 hour also increased HPV16 binding (1.37 fold) (p < 0.10 1-tail t test) (Fig. 4B). Maximal binding was observed with 1 hour of treatment with either protease resulting in a 30% increase in infectivity for cathepsin B treated reporter virions and 37% increase in infectivity for cathepsin L treated reporter-virions. Since binding increased by a third, the difference can be considered physiologically significant in addition to statistically significant. Binding was observed to decrease with cathepsin B and L treatments for more than 1 hour. These data suggest that protease treatment of HPV16 for 1 hour can increase binding, that longer time incubation results in a loss of total binding, and that the increase in infection after protease treatment of reporter-virions may not be a direct result of increased binding.

### Protease treatment of reporter-virions overcomes furin inhibition

**Figure 5:**
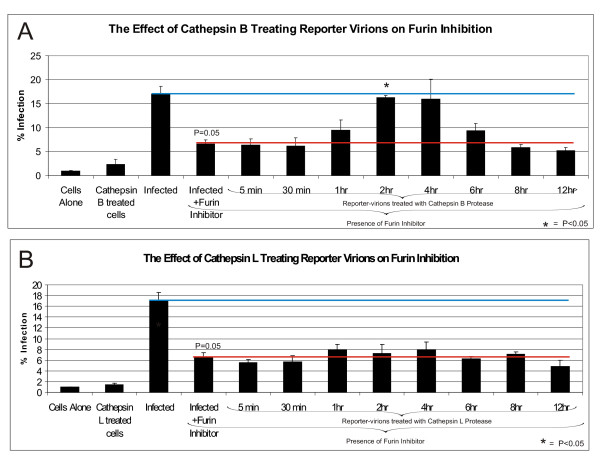
**Cathepsin B treatment overcomes block of infection caused by furin protease inhibition**. 293 cells incubated with 60 μM of furin inhibitor DEC-RVKR-CMK overnight were infected in the presence of mock treated (control), cathepsin B protease (A, 37 pM) or cathepsin L protease (B, 37 pM) treated reporter-virions for various amounts of time. Cells were analyzed 48 hours post binding by flow cytometry for the presence of DsRed expression. Statistics were analyzed by 1 tailed t-test and found to have a significant difference at P < 0.05.

BPV1 infection has been shown to be dependent on a furin cleavage event [2,14,24]. This proteolytic event is thought to occur after an initial conformational change in the viral capsid [24]. To determine if cysteine proteases played a role in mediating furin protease cleavage, we infected cells incubated with a furin inhibitor using cysteine protease treated reporter-virions. 293 cells were incubated with furin inhibitor overnight, infected with HPV16 reporter-virions that were treated for various amounts of time with cathepsin B or cathepsin L protease, and infections were analyzed by flow cytometry after 48 hours (for DsRED fluorescence). As previously described [24], furin inhibition decreased HPV16 infection by 61.39% compared to cells that were infected in the absence of the furin inhibitor (Fig. 5A, Infected and Infected + Furin Inhibitor bars). To our surprise, cathepsin B treated HPV16 reporter-virions for 2 and 4 hours had a significant increase in infection levels (p < 0.05 1-tail t test) in the presence of furin inhibitor as compared to infection of untreated reporter-virions in the presence of furin inhibitor (compare Fig. 5A 2 and 4 hr bars to infected + Furin inhibitor bar). Cathepsin B treated reporter-virions for 2 hours were as infectious in the presence of furin inhibitor as infected control without protease treatment or furin inhibition (Compare Fig 5A Infected and 2, 4 hr bars). Reporter-virions treated with cathepsin L protease were unable to overcome furin inhibition and were unable to reach levels comparable to that of the infected control without furin inhibition (Fig 5B) (p < 0.05 1-tail T test). These data suggested that cathepsin B but not cathepsin L treatment of HPV16 reporter-virions can overcome furin inhibition.

### HPV16 Infectivity is blocked in the absence of cathepsin B

**Figure 6:**
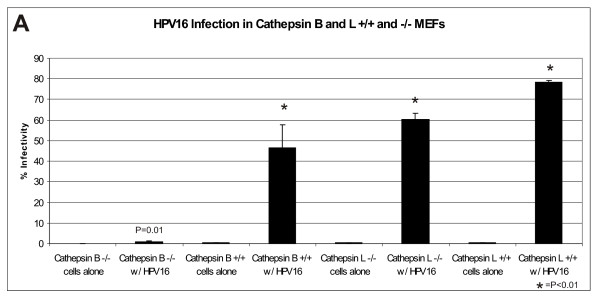
**Lack of HPV16 Infection in the absence of cathepsin B**. HPV16 infection levels in cathepsin B and cathepsin L deficient (-/-) and wild type (+/+) MEF cells were analyzed and compared 48 hours post binding by FACS analysis of GFP expression. Cells alone were analyzed for background fluorescence. Statistics were analyzed by 1 tailed t-test and found to have a significant difference at P < 0.05.

Because our data suggested that cathepsin B may play a role in the infection pathway of 293 cells, we were interested in determining if our observation was cell line specific. We performed infection experiments using cathepsin deficient mouse embryonic fibroblast (MEFs) cell lines. Using the cathepsin knockout MEFs we were able to confirm the role of cathepsins because unlike inhibitors which may have non-specific effects on the host cell and do not inhibit 100% of the target leading to partial effects in infectivity, knockout MEFs ensure that cathepsin B or L were completely absent. MEFs deficient for either cathepsin B, cathepsin L, or wild type MEFs, i.e., cathepsin B and L positive, were infected with HPV16 reporter-virions and analyzed for infection levels after 48 hours. Compared to the 46.58% infection in the cathepsin B +/+ wild type MEFs (Fig 6A, cath B +/+ with HPV16), cathepsin B deficient -/- MEFs were not susceptible to infection, yielding only 0.80% infection (Fig 6A, cath B -/- with HPV16). Cathepsin L wild type +/+ MEFs (Fig 6A, cath L +/+ with HPV16) showed an increase in infection levels (78.38%) compared to cathepsin L -/- deficient MEFs (Fig 6A, cath L -/- with HPV16) which had a 60.26% infection level. Cells alone showed no background levels of autofluorescence (Fig 6A cells alone bars). The data suggested that cathepsin B protease is required for infection of HPV16 in MEFs.

## Discussion

Cathepsin proteases have been shown to be able to modify the binding and entry of many enveloped viruses, and thus influence infection efficiency [31-36]. In this manuscript, we broadened the involvement of cathepsin proteases to the non-enveloped virus, HPV16. We showed that cathepsin proteases play a role in the infection of HPV16 into human embryonic 293 cells (HEK 293), and into mouse embryonic fibroblasts (MEFs).

Data previously obtained by our laboratory has demonstrated that BPV1 and HPV16 infection follow a 'non-classical' trafficking route post-clathrin-mediated endocytosis [3,4]. Our data demonstrated that after reporter-virions have endocytosed using clathrin-coated pits, the reporter-virions are found in an early endosome (EEA1 positive vesicle), and are sorted to a caveolin-1 positive organelle putatively the caveosome before colocalizing with endoplasmic reticulum (ER) marker. Caveosome were originally identified as a necessary component of SV-40 trafficking [38] and subsequent work has demonstrated that reporter-virions seem to traffic through caveosomes on their way to the endoplasmic reticulum. In order for BPV1 and HPV16 to reach the caveosome, there would have to be cross-talk between endosomes and caveosomes. This cross-talk has now been described for JC virus [1] and for HPV 31 [39]. A commonly used technique that is used to determine the role of pH and endo-lysosomes in viral trafficking involves using the lysosome pH neutralizing chemical ammonium chloride (NH_4_Cl) to prevent the acidification of vesicles, unfortunately this treatment also results in the loss of fusion of intracellular vesicles. In fact, it was shown that NH_4_Cl prevented the movement of JC virus from endosome to caveosome.

The observed loss of viral infection using NH_4_Cl posed two hypotheses: 1) that the PV infection was preventing the fusion of vesicles, and 2) that NH_4_Cl was preventing the function of endo-lysosome proteases by preventing their conversion from "pro-inactive" form to "active" form. Our data shown in Figure 2 confirmed that indeed there was loss of movement of HPV 16 reporter-virions from endosomes to caveosome that could account for the loss of infection observed using NH_4_Cl (shown in Fig 1). Regarding the role of endo-lysosome proteases, we focused on the cysteine proteases cathepsin B and L, two highly abundant proteases in the endo-lysosome compartments, and as mentioned above, cathepsin B and L have been previously shown to be involved in viral infections. Our data showed that broad cysteine protease inhibitors and specific cathepsin B or L inhibitors were able to decrease infection, thus suggesting that cysteine proteases were in part mediating HPV16 infection in 293 cells.

Furin protease has been shown to be necessary for viral infection by allowing the escape of the viral particle from an endosome (Richards RM, Lowy DR, Schiller JT, Day PM [24]). Richards and colleagues theorized that furin allowed the escape of reporter-virions from an endosome as observed by the loss of endosome marker (EEA1) staining overlap with reporter-virions. Our data supports a loss of EEA1 overlap with reporter-virions but show that reporter-virions are moved to a caveolin-1 vesicle. It is unclear where Richards and colleagues propose viral particles escape to. Because furin cleavage was shown to occur after capsid conformation changes, we addressed if cathepsins B or L were playing a role in capsid structural changes that aided the furin cleavage event. To our surprise the pre-treatment of purified viral particles with cathepsin B, but not cathepsin L, was able to overcome the block of infection observed in the presence of furin inhibitor. The significance of this finding needs further work. It is possible that HPV16 utilizes cathepsin B as a "backup" mechanism for furin in order to establish infection.

In a recent study addressing the role of endosome proteases in the disassembly of HPV16 [24], the authors negated the role of cathepsin B and L in the HPV16 infectious process. The differences between our studies and theirs may be due to the variation in inhibitors, cell lines, and quantity of viral particles used. In the paper by Richards and colleagues the cathepsin B inhibitor used was CA-074, a membrane impermeable inhibitor that would not have shown an effect on an intracellular process (we used both a permeable and non-permeable inhibitor); the cathepsin L inhibitor used was only described as "cathepsin L inhibitor" and no further conclusions can be drawn as to the specificity of the inhibitor [24]. In addition, the observations described by Richards and colleagues were seen in the HPV 18 positive cervical carcinoma HeLa cells while our studies were performed in the adenovirus E1A transformed human embryonic kidney 293 cell line and in MEFs derived from cathepsin B-deficient mice and cathepsin L-deficient mice [24]. HeLa cells were recently shown to be infected by a non-clathrin mediated endocytosis event, a finding that may also explain the differences in both studies [40]. Finally the experiments performed in the paper by Richards and colleagues, used a minimum of 7.5 ng of PV while our experiments were carried out using less than 0.33 ng of HPV16 (1 ng of VLPs has 30 million particles); a difference that may also contribute to the differences in results.

## Conclusion

In summary, our data in this manuscript supports that during the process of infection, HPV16 is subjected to partial proteolysis by cathepsins at the plasma membrane, in the endo-lysosome, or in the caveolin-1 positive vesicles. Work pursuing the role of cysteine proteases in HaCaT keratinocytes and the specific biological significance and cellular site of cathepsin proteolysis is on-going.

## Methods

### Cells, antibodies, proteases and inhibitors

293 cells, a human embryonic kidney cell line (HEK), HaCaT cells, spontaneously immortalized human keratinocytes, and cathepsin B deficient (-/-), wild type (+/+), cathepsin L deficient (-/-) and wild type (+/+) mouse embryonic fibroblasts (MEFs) were grown in Dulbecco's Modified Eagle's medium (DMEM) supplemented with 10% Fetal Bovine Serum (DMEM-10), and 100 IU/mL penicillin/streptomycin. MEF cells were gifts from Dr. T. S. Dermody (Vanderbilt University School of Medicine, Nashville, TN). Goat anti-EEA1 (which recognizes early endosomes) antibody was purchased from Santa Cruz Biotechnology, Santa Cruz, CA. Rabbit anti-caveolin 1 antibody was purchased from Cell Signaling Technology (Danvers, MA). The monoclonal HPV16 L1 antibody, H16 V.5 was a generous gift from Dr. Neil Christensen (Penn State University, Hershey, PA). The following inhibitors were obtained from Calbiochem (Gibbstown, NJ) and used at the following non-toxic concentration: CA-074Me (6 μM), Z-FF-FMK an irreversible cell permeable cathepsin L inhibitor (10 μM), E64 (10 μM), E64-d (10 μM). Furin inhibitor DEC-RVKR-CMK was obtained from Biomol International (Plymouth Meeting, PA) and used at the non-toxic concentration of 60 μM. Ammonium chloride was used at a 20 mM concentration as per Day et al., (Sigma, St. Louis, MO) [2]. Cathepsin B protease (Calbiochem) and cathepsin L protease (R&D Systems Minneapolis, MN) were used at a concentration of 37 pM, a lower concentration compared to Ebert et al., [36].

### Flow cytometry

Inhibitors were added to 293 cells and allowed to incubate on cells at 37°C with 5% CO_2 _for 2 hours prior to infection. After the 2 hr incubation period, the cells were placed on ice. HPV 16 reporter-virions containing a DsRed or GFP transgene were added to cells on ice for 2 hours to allow for binding. At 2 hours, inhibitors and unbound virus were removed by washing with DMEM-10 and replaced with 500 μl of warm DMEM-10 plus inhibitor. MEFs (not treated with inhibitors) were placed on ice for 2 hours in the presence of HPV16 reporter-virions to allow for binding. Unbound reporter-virions were removed by washing with DMEM-10 and replaced with 500 μl of warm DMEM-10. The cells were incubated at 37°C with 5% CO_2 _for 48 hours. Cells were harvested using trypsin. The cells were spun for 1 min at 16,100 × G, the pellet was washed 5× in 1× PBS and resuspended in 300 ul of 1× PBS. 10,000 cells were counted on a fluorescence activated cell sorter (FACS) and the number of DsRed or GFP positive cells was used to determine the percent of infected cells (FACS performed at RFUMS Flow cytometry core). All experiments were repeated using reporter-virions made from different preparations.

### Immunofluorescence

Cells transfected on coverslips were washed 3× in 1× PBS and fixed in 4% paraformaldehyde for 20 min at 4°C. Paraformaldehyde was removed via 3 washes with 1× PBS. Cells were permeabilized with blocking buffer (0.2% fish skin gelatin (Sigma) and 0.2% Triton X-100 in PBS) for 5 min. The coverslips were washed 3× with 1× PBS and incubated with the appropriate primary antibody at 1:100 dilution in blocking buffer (1:25 dilution for LAMP1 antibody). Fluorescence labelled Alexa-flour donkey anti-mouse 488, goat anti-rabbit 594, chicken anti-goat 594, (Molecular Probes/Invitrogen, Eugene, OR) were used as secondary antibodies at 1:2,000 dilution in blocking buffer in a 30 minute incubation. Coverslips were incubated for 5 minutes with TOPRO-3 (Invitrogen, Carlsbad CA) at 1:1000 dilution for nuclear staining. The coverslips were mounted on glass slides using Prolong anti-fade mounting medium (Invitrogen). Fluorescence confocal microscopy and stereology (the quantification of the percentage of colocalization observed in the image, i.e. merged colors) were performed using an Olympus Fluoview 300 microscope, and analyzed with Fluoview and stereology software (Olympus, Melville, NY) at the microscopy core of Rosalind Franklin University of Medicine and Science (RFUMS) (North Chicago, IL). All images are shown with z stacks.

### Cytotoxicity Assay

Cytotoxicity studies for the various inhibitors were carried out using the CellTiter-Glo Luminescent Cell Viability Assay Kit (Promega, Madison, WI). Cells were incubated with various concentrations of inhibitors for 48 hours and then the supernatant and trypsinized cells were collected. 100 μl of the harvested cell suspension was added to a well in a 96-well plate. The CellTiter-Glo substrate and buffer were combined, and 100 μl was added to each well containing sample. The reagent and cells are mixed for 5 min on a shaker at room temperature. The 96-well plate was then allowed to rest at room temperature for 10 minutes before being analyzed by a Bio-Tek Synergy HT Plate Reader using the KC4 V3.4 software (Bio-Tek, Winooski, VT). All samples were analyzed in triplicate.

### Reporter-virion production and purification

Reporter-virions were made as described [37]. In brief, 293TT cells were co-transfected with p16llwcha, a bicistronic HPV16 L1 and L2 plasmid and 8frb, the DsRed or 8fwb, the GFP cDNA containing packaging plasmid. Constructs and cells were gifts from Drs. Day and Schiller (National Cancer Institute, National Institute of Health, Bethesda, MD). Cells were harvested and lysed after 48 hours. Reporter-virions were allowed to mature at 37°C over night allowing for proper conformation of the capsid proteins. After a high salt extraction, reporter-virions were purified on an optiprep gradient (27%–39%) via ultracentrifugation. Titer of reporter-virions was determined by FACS for the percentage of DsRed or GFP positive cells 48 hours after infection. Tritium labelled reporter-virions were made with the addition of ^3^H 24 hours post transfection.

### Binding of radioactive reporter-virions

Tritium labelled reporter-virions containing a DsRed transgene were added to 293 cells on ice for 2 hours to allow for binding without internalization. The unbound reporter-virions were removed by washing with 1× PBS. Cells were harvested in 30 μl 1× PBS, spotted on Whatmann paper and allowed to dry. The samples were fixed in 5% Trichloroacetic acid (TCA) for 20 minutes and precipitated in 95% ethanol for 20 minutes. The samples were analyzed with the LS 6500 Multipurpose Scintillation Counter (Beckman Coulter, Palatine, IL). All experiments were repeated using reporter-virions made from different preparations.

## Competing interests

The authors declare that they have no competing interests.

## Authors' contributions

SAD carried out the flow cytometric analysis, immunofluorescence analysis, radioactive studies, cytotoxicity analysis, and performed the statistical analysis. PIM and SAD participated in the design of the study and drafted the manuscript. All authors read and approved the final manuscript.

## Supplementary Material

Additional file 1 **Cytotoxicity of Inhibitors in 293 cells**. 293 cells where incubated with various concentrations of a non-permeable cysteine protease inhibitor E64 (A), the permeable cysteine protease inhibitor E64-d (B), cathepsin L inhibitor (C), the intracellular cathepsin B inhibitor CA074-ME (D), furin inhibitor (E) and the lysosome neutralizing agent NH_4_Cl (F) for 48 hours. The cells and supernatant were analyzed for cytotoxicity using a plate reader. Cells alone were analyzed for background fluorescence. Click here for file

Additional file 2 **Dose Dependent Concentration of Inhibitors in 293 cells**. 293 cells where incubated with various concentrations of a non-permeable cysteine protease inhibitor E64 (A), the permeable cysteine protease inhibitor E64-d (B), cathepsin L inhibitor (C), the intracellular cathepsin B inhibitor CA074-ME (D), furin inhibitor (E) and the lysosome neutralizing agent NH_4_Cl (F) overnight. The cells were then infected in the presence of inhibitor with HPV16 reporter-virions containing a GFP reporter gene. The cells were analyzed and compared 48 hours post binding by FACS analysis of GFP expression. Cells alone were analyzed for background fluorescence. Statistics were analyzed by 1 tailed t-test and found to have a significant difference at P < 0.05. Click here for file

Additional file 3 **HPV16 infection is reduced in the presence of a cysteine protease inhibitor in HaCat cells**. Infection of HaCat cells alone, with HPV 16 GFP reporter-virions (mock control, Infected), or in the presence of: 20 mM of lysosome neutralizing agent NH_4_Cl or 10 μM of a non-permeable cysteine protease inhibitor E64. Infection was analyzed and compared 48 hours post binding by FACS of GFP expression. Inhibitors were present for the duration of infection. Cells alone were analyzed for background fluorescence. Statistics were analyzed by 1 tailed t-test and found to be significant at P < 0.05. Click here for file

Additional file 4 **NH_4 _Cl does not decrease HPV16 reporter-virions ability to infect**. HPV16 GFP reporter-virions were incubated with (A) 20 mM NH_4_Cl for various amounts of time. 293 cells were infected with NH_4_Cl treated reporter-virions or infected with untreated reporter-virions (control). Infections were analyzed by flow cytometry for GFP expression 48 hours post binding. 293 cells alone were used as control for background fluorescence. Statistics were analyzed by 1 tailed t-test and found be significant at P < 0.05. Click here for file

Additional file 5 **Additional Incubation of HPV16 reporter-virions at 37°C does not increase infectivity**. HPV16 reporter-virions containing a GFP reporter gene were incubated at 37°C for various amounts of time. 293 cells were infected with the HPV16 reporter-virions and analyzed by flow cytometry for GFP expression 48 hours post binding. Statistics were analyzed by 1 tailed t-test and found to be significant at P < 0.05. Click here for file
