# Supplementary figures and images for: The role of NH4Cl and cysteine proteases in Human Papillomavirus type 16 infection
Source: Virol J. 2009 Jul 20;6:109. doi: 10.1186/1743-422X-6-109 (PMC2718874; doi:10.1186/1743-422X-6-109)

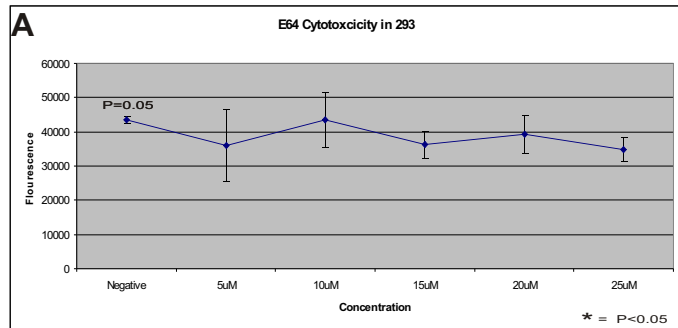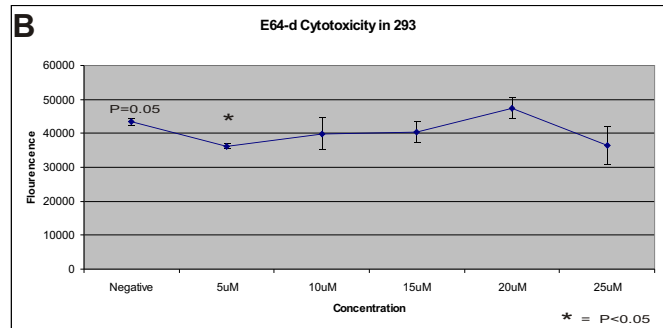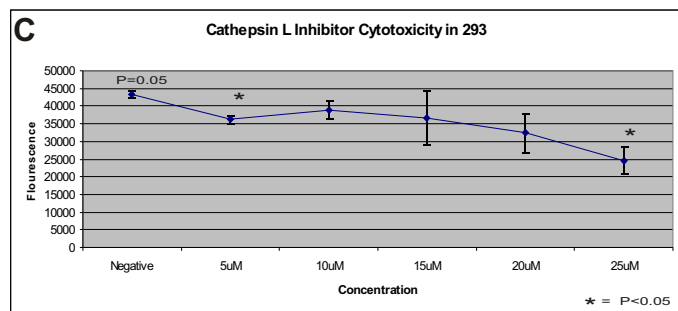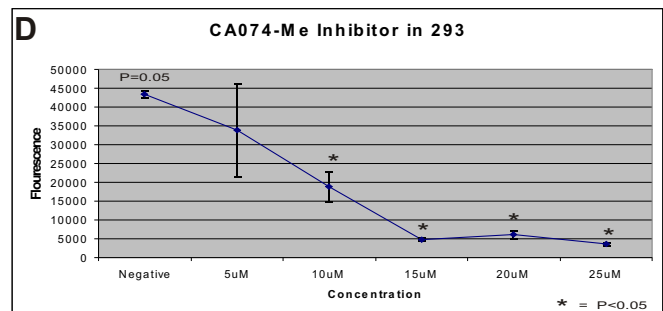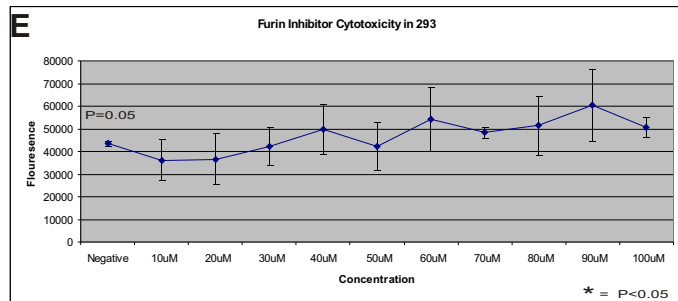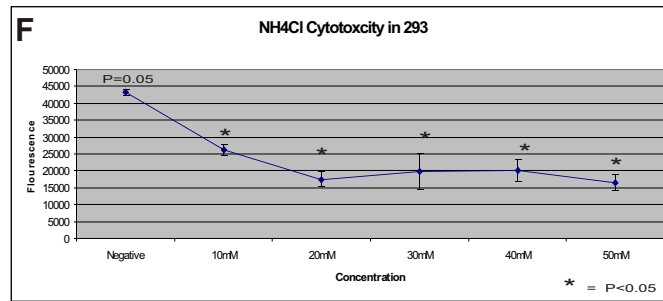

Supplement: Additional file 1 — Cytotoxicity of Inhibitors in 293 cells. 293 cells where incubated with various concentrations of a non-permeable cysteine protease inhibitor E64 (A), the permeable cysteine protease inhibitor E64-d (B), cathepsin L inhibitor (C), the intracellular cathepsin B inhibitor CA074-ME (D), furin inhibitor (E) and the lysosome neutralizing agent NH4Cl (F) for 48 hours. The cells and supernatant were analyzed for cytotoxicity using a plate reader. Cells alone were analyzed for background fluorescence. [file 1743-422X-6-109-S1.pdf]

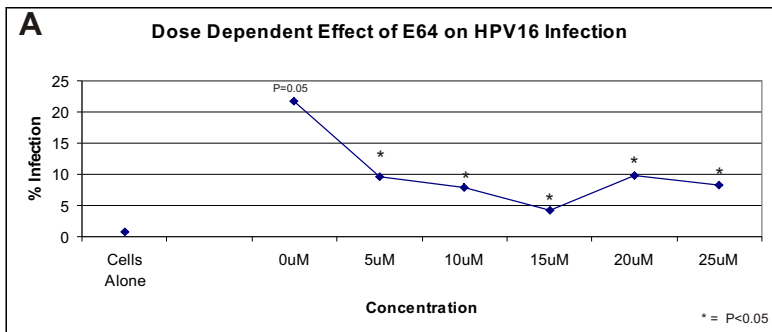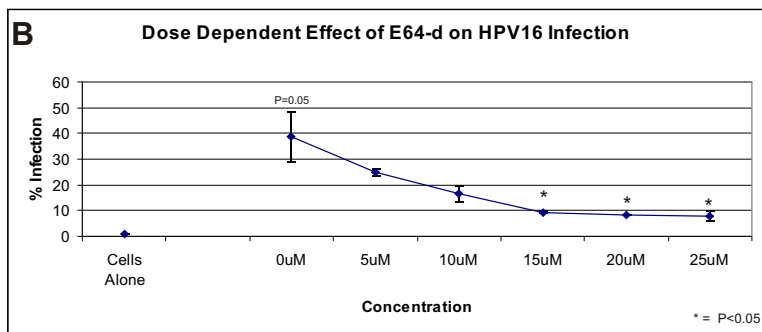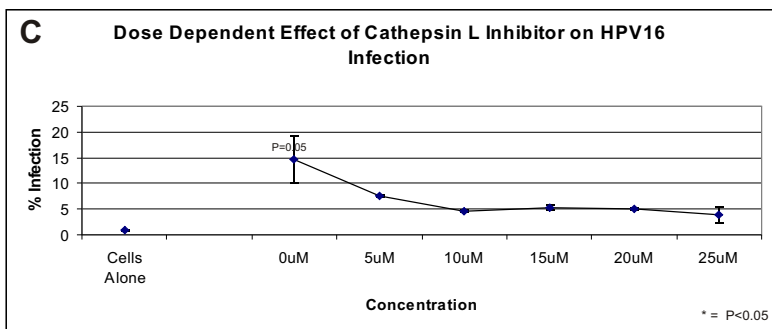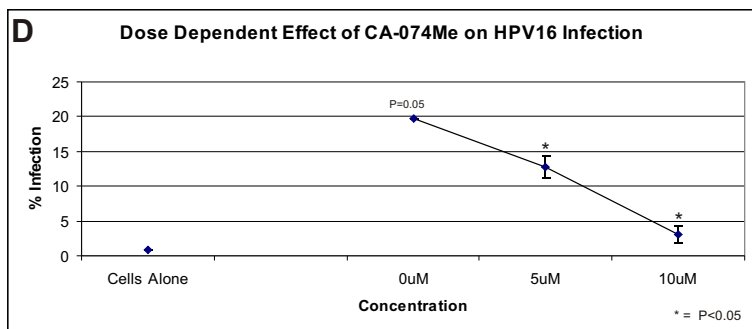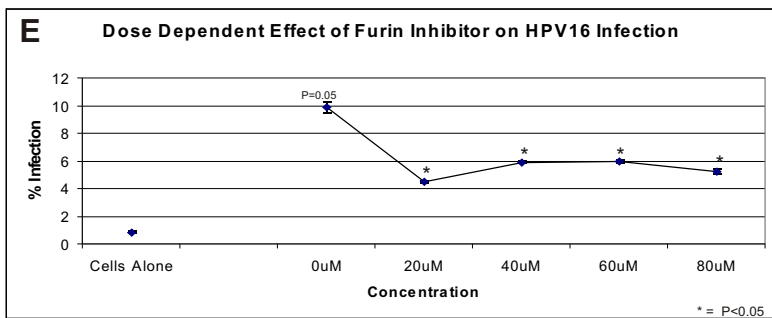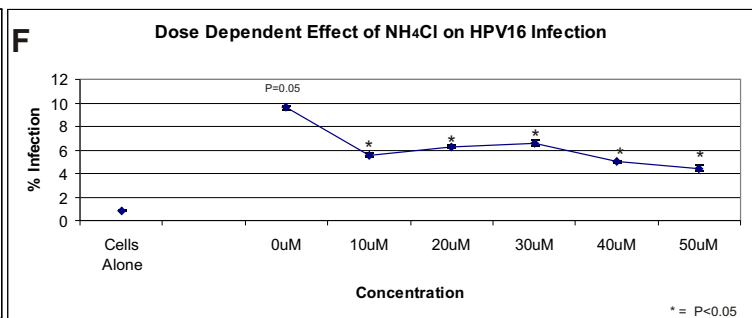

Supplement: Additional file 2 — Dose Dependent Concentration of Inhibitors in 293 cells. 293 cells where incubated with various concentrations of a non-permeable cysteine protease inhibitor E64 (A), the permeable cysteine protease inhibitor E64-d (B), cathepsin L inhibitor (C), the intracellular cathepsin B inhibitor CA074-ME (D), furin inhibitor (E) and the lysosome neutralizing agent NH4Cl (F) overnight. The cells were then infected in the presence of inhibitor with HPV16 reporter-virions containing a GFP reporter gene. The cells were analyzed and compared 48 hours post binding by FACS analysis of GFP expression. Cells alone were analyzed for background fluorescence. Statistics were analyzed by 1 tailed t-test and found to have a significant difference at P < 0.05. [file 1743-422X-6-109-S2.pdf]

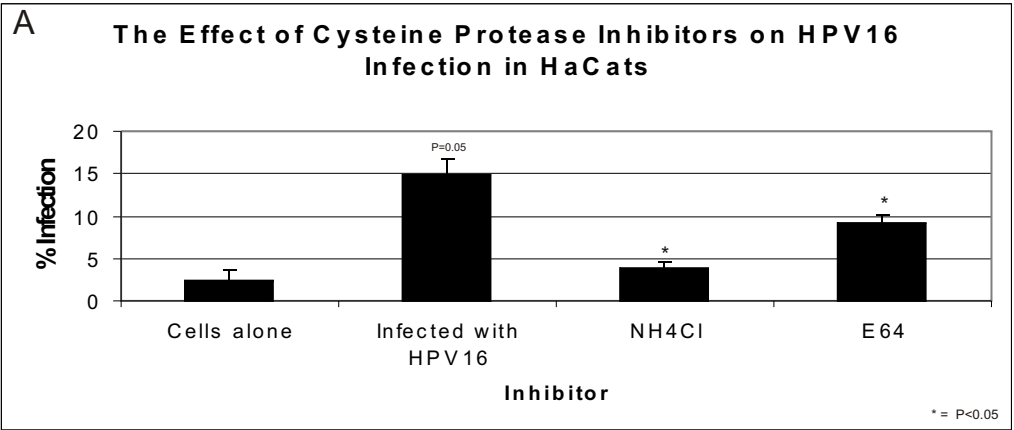

Supplement: Additional file 3 — HPV16 infection is reduced in the presence of a cysteine protease inhibitor in HaCat cells. Infection of HaCat cells alone, with HPV 16 GFP reporter-virions (mock control, Infected), or in the presence of: 20 mM of lysosome neutralizing agent NH4Cl or 10 μM of a non-permeable cysteine protease inhibitor E64. Infection was analyzed and compared 48 hours post binding by FACS of GFP expression. Inhibitors were present for the duration of infection. Cells alone were analyzed for background fluorescence. Statistics were analyzed by 1 tailed t-test and found to be significant at P < 0.05. [file 1743-422X-6-109-S3.pdf]

A

# The Effect of NH<sub>4</sub>Cl on HPV16 Reporter-virions

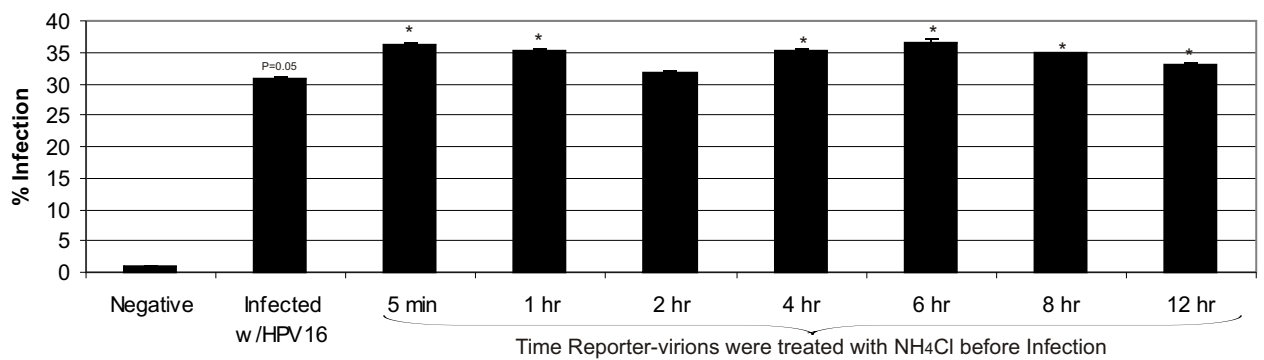

\*

Supplement: Additional file 4 — NH4 Cl does not decrease HPV16 reporter-virions ability to infect. HPV16 GFP reporter-virions were incubated with (A) 20 mM NH4Cl for various amounts of time. 293 cells were infected with NH4Cl treated reporter-virions or infected with untreated reporter-virions (control). Infections were analyzed by flow cytometry for GFP expression 48 hours post binding. 293 cells alone were used as control for background fluorescence. Statistics were analyzed by 1 tailed t-test and found be significant at P < 0.05. [file 1743-422X-6-109-S4.pdf]

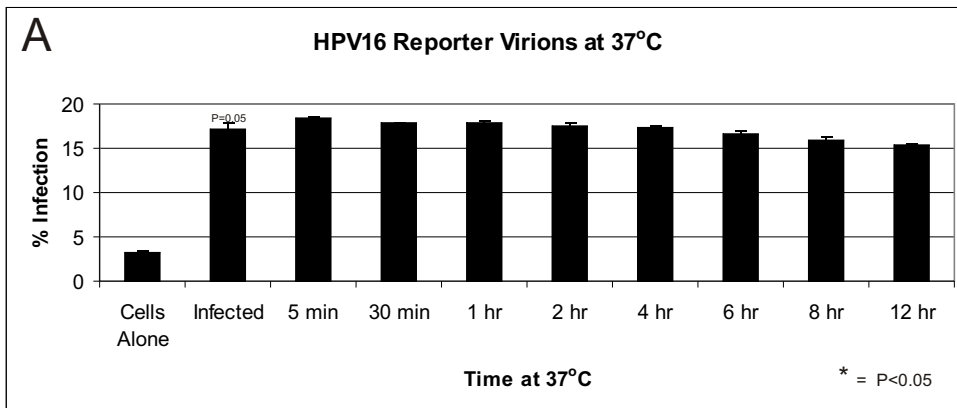

Supplement: Additional file 5 — Additional Incubation of HPV16 reporter-virions at 37°C does not increase infectivity. HPV16 reporter-virions containing a GFP reporter gene were incubated at 37°C for various amounts of time. 293 cells were infected with the HPV16 reporter-virions and analyzed by flow cytometry for GFP expression 48 hours post binding. Statistics were analyzed by 1 tailed t-test and found to be significant at P < 0.05. [file 1743-422X-6-109-S5.pdf]
